# Solve-RD: systematic pan-European data sharing and collaborative analysis to solve rare diseases

**DOI:** 10.1038/s41431-021-00859-0

**Published:** 2021-06-01

**Authors:** Birte Zurek, Kornelia Ellwanger, Lisenka E. L. M. Vissers, Rebecca Schüle, Matthis Synofzik, Ana Töpf, Richarda M. de Voer, Steven Laurie, Leslie Matalonga, Christian Gilissen, Stephan Ossowski, Peter A. C. ’t Hoen, Antonio Vitobello, Julia M. Schulze-Hentrich, Olaf Riess, Han G. Brunner, Anthony J. Brookes, Ana Rath, Gisèle Bonne, Gulcin Gumus, Alain Verloes, Nicoline Hoogerbrugge, Teresinha Evangelista, Tina Harmuth, Morris Swertz, Dylan Spalding, Alexander Hoischen, Sergi Beltran, Holm Graessner, Solve-RD consortium, Tobias B. Haack, Birte Zurek, Kornelia Ellwanger, German Demidov, Marc Sturm, Christoph Kessler, Melanie Wayand, Carlo Wilke, Andreas Traschütz, Ludger Schöls, Holger Hengel, Peter Heutink, Han Brunner, Hans Scheffer, Wouter Steyaert, Karolis Sablauskas, Richarda M. de Voer, Erik-Jan Kamsteeg, Bart van de Warrenburg, Nienke van Os, Iris te Paske, Erik Janssen, Elke de Boer, Marloes Steehouwer, Burcu Yaldiz, Tjitske Kleefstra, Colin Veal, Spencer Gibson, Marc Wadsley, Mehdi Mehtarizadeh, Umar Riaz, Greg Warren, Farid Yavari Dizjikan, Thomas Shorter, Volker Straub, Chiara Marini Bettolo, Sabine Specht, Jill Clayton-Smith, Siddharth Banka, Elizabeth Alexander, Adam Jackson, Laurence Faivre, Christel Thauvin, Antonio Vitobello, Anne-Sophie Denommé-Pichon, Yannis Duffourd, Emilie Tisserant, Ange-Line Bruel, Christine Peyron, Aurore Pélissier, Sergi Beltran, Ivo Glynne Gut, Steven Laurie, Davide Piscia, Leslie Matalonga, Anastasios Papakonstantinou, Gemma Bullich, Alberto Corvo, Carles Garcia, Marcos Fernandez-Callejo, Carles Hernández, Daniel Picó, Ida Paramonov, Hanns Lochmüller, Gulcin Gumus, Virginie Bros-Facer, Marc Hanauer, Annie Olry, David Lagorce, Svitlana Havrylenko, Katia Izem, Fanny Rigour, Giovanni Stevanin, Alexandra Durr, Claire-Sophie Davoine, Léna Guillot-Noel, Anna Heinzmann, Giulia Coarelli, Valérie Allamand, Isabelle Nelson, Rabah Ben Yaou, Corinne Metay, Bruno Eymard, Enzo Cohen, Antonio Atalaia, Tanya Stojkovic, Milan Macek, Marek Turnovec, Dana Thomasová, Radka Pourová Kremliková, Vera Franková, Markéta Havlovicová, Vlastimil Kremlik, Helen Parkinson, Thomas Keane, Alexander Senf, Peter Robinson, Daniel Danis, Glenn Robert, Alessia Costa, Christine Patch, Mike Hanna, Henry Houlden, Mary Reilly, Jana Vandrovcova, Francesco Muntoni, Irina Zaharieva, Anna Sarkozy, Vincent Timmerman, Jonathan Baets, Liedewei Van de Vondel, Danique Beijer, Peter de Jonghe, Vincenzo Nigro, Sandro Banfi, Annalaura Torella, Francesco Musacchia, Giulio Piluso, Alessandra Ferlini, Rita Selvatici, Rachele Rossi, Marcella Neri, Stefan Aretz, Isabel Spier, Anna Katharina Sommer, Sophia Peters, Carla Oliveira, Jose Garcia Pelaez, Ana Rita Matos, Celina São José, Marta Ferreira, Irene Gullo, Susana Fernandes, Luzia Garrido, Pedro Ferreira, Fátima Carneiro, Morris A. Swertz, Lennart Johansson, Joeri K. van der Velde, Gerben van der Vries, Pieter B. Neerincx, Dieuwke Roelofs-Prins, Sebastian Köhler, Alison Metcalfe, Alain Verloes, Séverine Drunat, Caroline Rooryck, Aurelien Trimouille, Raffaele Castello, Manuela Morleo, Michele Pinelli, Alessandra Varavallo, Manuel Posada De la Paz, Eva Bermejo Sánchez, Estrella López Martín, Beatriz Martínez Delgado, F. Javier Alonso García de la Rosa, Andrea Ciolfi, Bruno Dallapiccola, Simone Pizzi, Francesca Clementina Radio, Marco Tartaglia, Alessandra Renieri, Elisa Benetti, Peter Balicza, Maria Judit Molnar, Ales Maver, Borut Peterlin, Alexander Münchau, Katja Lohmann, Rebecca Herzog, Martje Pauly, Alfons Macaya, Anna Marcé-Grau, Andres Nascimiento Osorio, Daniel Natera de Benito, Hanns Lochmüller, Rachel Thompson, Kiran Polavarapu, David Beeson, Judith Cossins, Pedro M. Rodriguez Cruz, Peter Hackman, Mridul Johari, Marco Savarese, Bjarne Udd, Rita Horvath, Gabriel Capella, Laura Valle, Elke Holinski-Feder, Andreas Laner, Verena Steinke-Lange, Evelin Schröck, Andreas Rump

**Affiliations:** 1 Institute of Medical Genetics and Applied Genomics, University of Tübingen, Tübingen, Germany; 2 Department of Human Genetics, Radboud University Medical Center, Nijmegen, The Netherlands; 3 Donders Institute for Brain, Cognition and Behaviour, Radboud University Medical Center, Nijmegen, The Netherlands; 4 Department of Neurodegeneration, Hertie Institute for Clinical Brain Research (HIH), University of Tübingen, Tübingen, Germany; 5 German Center for Neurodegenerative Diseases (DZNE), Tübingen, Germany; 6 John Walton Muscular Dystrophy Research Centre, Translational and Clinical Research Institute, Newcastle University and Newcastle Hospitals NHS Foundation Trust, Newcastle upon Tyne, UK; 7 Radboud Institute for Molecular Life Sciences, Nijmegen, The Netherlands; 8 CNAG-CRG, Centre for Genomic Regulation (CRG), The Barcelona Institute of Science and Technology, Barcelona, Spain; 9 Center for Molecular and Biomolecular Informatics, Radboud University Medical Center, Nijmegen, The Netherlands; 10 Inserm—University of Burgundy-Franche Comté, Dijon, France; 11 Centre for Rare Diseases, University of Tübingen, Tübingen, Germany; 12 Department of Clinical Genetics, Maastricht University Medical Centre, Maastricht, The Netherlands; 13 Department of Genetics and Genome Biology, University of Leicester, Leicester, UK; 14 INSERM, US14—Orphanet, Plateforme Maladies Rares, Paris, France; 15 Sorbonne Université, INSERM UMRS 974, Center of Research in Myology, Paris, France; 16 EURORDIS-Rare Diseases Europe, Barcelona, Spain; 17 Genetics Department, APHP-Robert Debré University Hospital, Université de Paris, Paris, France; 18 Department of Genetics, Genomics Coordination Center, University Medical Center Groningen, University of Groningen, Groningen, The Netherlands; 19 European Bioinformatics Institute, European Molecular Biology Laboratory, Wellcome Genome Campus, Hinxton, Cambridge, UK; 20 Department of Internal Medicine and Radboud Center for Infectious Diseases (RCI), Radboud University Medical Center, Nijmegen, The Netherlands; 21 Universitat Pompeu Fabra (UPF), Barcelona, Spain; 22 Departament de Genètica, Microbiologia i Estadística, Facultat de Biologia, Universitat de Barcelona (UB), Barcelona, Spain; 23 Department of Neurology, Radboud University Medical Center, Nijmegen, The Netherlands; 24 Division of Evolution and Genomic Sciences, School of Biological Sciences, Faculty of Biology, Medicine and Health, University of Manchester, Manchester, UK; 25 Manchester Centre for Genomic Medicine, St Mary’s Hospital, Manchester University Hospitals NHS Foundation Trust, Health Innovation Manchester, Manchester, UK; 26 Dijon University Hospital, Genetics Department, Dijon, France; 27 Dijon University Hospital, Centre of Reference for Rare Diseases, Development Disorders and Malformation Syndromes, Dijon, France; 28 Dijon University Hospital, FHU-TRANSLAD, Dijon, France; 29 Dijon University Hospital, GIMI Institute, Dijon, France; 30 University of Burgundy-Franche Comté, Dijon Economics Laboratory, Dijon, France; 31 University of Burgundy-Franche Comté, FHU-TRANSLAD, Dijon, France; 32 EURORDIS-Rare Diseases Europe, Sant Antoni Maria Claret 167 - 08025, Barcelona, Spain; 33 EURORDIS-Rare Diseases Europe, Plateforme Maladies Rares, Paris, France; 34 Institut National de la Santé et de la Recherche Medicale (INSERM) U1127, Paris, France; 35 Centre National de la Recherche Scientifique, Unité Mixte de Recherche (UMR) 7225, Paris, France; 36 Unité Mixte de Recherche en Santé 1127, Université Pierre et Marie Curie (Paris 06), Sorbonne Universités, Paris, France; 37 Institut du Cerveau -ICM, Paris, France; 38 Ecole Pratique des Hautes Etudes, Paris Sciences et Lettres Research University, Paris, France; 39 Centre de Référence de Neurogénétique, Hôpital de la Pitié-Salpêtrière, Assistance Publique-Hôpitaux de Paris (AP-HP), Paris, France; 40 Hôpital de la Pitié-Salpêtrière, Assistance Publique-Hôpitaux de Paris (AP-HP), Paris, France; 41 AP-HP, Centre de Référence de Pathologie Neuromusculaire Nord, Est, Ile-de-France, Institut de Myologie, G.H. Pitié-Salpêtrière, Paris, France; 42 Institut de Myologie, Equipe Bases de données, G.H. Pitié-Salpêtrière, Paris, France; 43 AP-HP, Unité Fonctionnelle de Cardiogénétique et Myogénétique Moléculaire et Cellulaire, G.H. Pitié-Salpêtrière, Paris, France; 44 Department of Biology and Medical Genetics, Charles University Prague-2nd Faculty of Medicine and University Hospital Motol, Prague, Czech Republic; 45 Jackson Laboratory for Genomic Medicine, Farmington, CT USA; 46 Florence Nightingale Faculty of Nursing and Midwifery, King’s College, London, UK; 47 Genetic Counselling, Genomics England, Queen Mary University of London, Dawson Hall, London, UK; 48 MRC Centre for Neuromuscular Diseases and National Hospital for Neurology and Neurosurgery, UCL Queen Square Institute of Neurology, London, UK; 49 Department of Neuromuscular Diseases, UCL Queen Square Institute of Neurology, London, UK; 50 Dubowitz Neuromuscular Centre, UCL Great Ormond Street Hospital, London, UK; 51 NIHR Great Ormond Street Hospital Biomedical Research Centre, London, UK; 52 Peripheral Neuropathy Research Group, Department of Biomedical Sciences, University of Antwerp, Antwerp, Belgium; 53 Institute Born Bunge, Antwerp, Belgium; 54 Peripheral Neuropathy Research Group, University of Antwerp, Antwerp, Belgium; 55 Neuromuscular Reference Centre, Department of Neurology, Antwerp University Hospital, Antwerpen, Belgium; 56 Laboratory of Neuromuscular Pathology, Institute Born-Bunge, University of Antwerp, Antwerpen, Belgium; 57 Dipartimento di Medicina di Precisione, Università degli Studi della Campania “Luigi Vanvitelli,”, Napoli, Italy; 58 Telethon Institute of Genetics and Medicine, Pozzuoli, Italy; 59 Unit of Medical Genetics, Department of Medical Sciences, University of Ferrara, Ferrara, Italy; 60 Institute of Human Genetics, University of Bonn, Bonn, Germany; 61 Center for Hereditary Tumor Syndromes, University Hospital Bonn, Bonn, Germany; 62 i3S - Instituto de Investigação e Inovação em Saúde, Universidade do Porto, Porto, Portugal; 63 IPATIMUP - Institute of Molecular Pathology and Immunology of the University of Porto, Porto, Portugal; 64 Department of Pathology, Faculty of Medicine, University of Porto, Porto, Portugal; 65 Department of Genetics, Faculty of Medicine, University of Porto, Porto, Portugal; 66 CHUSJ, Centro Hospitalar e Universitário de São João, Porto, Portugal; 67 Faculty of Sciences, University of Porto, Porto, Portugal; 68 NeuroCure Cluster of Excellence, Charité Universitätsklinikum, Charitéplatz 1, Berlin, Germany; 69 College of Health, Well-being and Life-Sciences, Sheffield Hallam University, Sheffield, UK; 70 Department of Genetics, Assistance Publique-Hôpitaux de Paris - Université de Paris, Robert DEBRE University Hospital, 48 bd SERURIER, Paris, France; 71 INSERM UMR 1141 “NeuroDiderot”, Hôpital R DEBRE, Paris, France; 72 Univ. Bordeaux, MRGM INSERM U1211, CHU de Bordeaux, Service de Génétique Médicale, Bordeaux, France; 73 Laboratoire de Génétique Moléculaire, Service de Génétique Médicale, CHU Bordeaux – Hôpital Pellegrin, Place Amélie Raba Léon, Bordeaux Cedex, France; 74 Institute of Rare Diseases Research, Spanish Undiagnosed Rare Diseases Cases Program (SpainUDP) & Undiagnosed Diseases Network International (UDNI), Instituto de Salud Carlos III, Madrid, Spain; 75 Genetics and Rare Diseases Research Division, Ospedale Pediatrico Bambino Gesù, IRCCS, Rome, Italy; 76 Med Biotech Hub and Competence Center, Department of Medical Biotechnologies, University of Siena, Siena, Italy; 77 Medical Genetics, University of Siena, Siena, Italy; 78 Genetica Medica, Azienda Ospedaliero-Universitaria Senese, Siena, Italy; 79 Institute of Genomic Medicine and Rare Diseases, Semmelweis University, Budapest, Hungary; 80 Clinical institute of genomic medicine, University medical centre Ljubljana, Ljubljana, Slovenia; 81 Institute of Neurogenetics, University of Lübeck, Lübeck, Germany; 82 Neurology Research Group, Vall d’Hebron Research Institute, Universitat Autònoma de Barcelona, Barcelona, Spain; 83 Neuromuscular Disorders Unit, Department of Pediatric Neurology. Hospital Sant Joan de Déu, Barcelona, Spain; 84 Department of Neuropediatrics and Muscle Disorders, Medical Center, Faculty of Medicine, University of Freiburg, Freiburg, Germany; 85 Centro Nacional de Análisis Genómico (CNAG-CRG), Center for Genomic Regulation, Barcelona Institute of Science and Technology (BIST), Barcelona, Spain; 86 Children’s Hospital of Eastern Ontario Research Institute, University of Ottawa, Ottawa, ON Canada; 87 Nuffield Department of Clinical Neurosciences, University of Oxford, Oxford, UK; 88 Folkhälsan Research Centre and Medicum, University of Helsinki, Helsinki, Finland; 89 Tampere Neuromuscular Center, Tampere, Finland; 90 Vasa Central Hospital, Vaasa, Finland; 91 Department of Clinical Neurosciences, University of Cambridge, Cambridge, UK; 92 Bellvitge Biomedical Research Institute (IDIBELL), Barcelona, Spain; 93 Medical Genetics Center (MGZ), Munich, Germany; 94 Institute for Clinical Genetics, Faculty of Medicine Carl Gustav Carus, Technical University Dresden, Dresden, Germany; 95 Center for Personalized Oncology, University Hospital Carl Gustav Carus, Technical University Dresden, Dresden, Germany

**Keywords:** Medical genetics, Diseases

## Abstract

For the first time in Europe hundreds of rare disease (RD) experts team up to actively share and jointly analyse existing patient’s data. Solve-RD is a Horizon 2020-supported EU flagship project bringing together >300 clinicians, scientists, and patient representatives of 51 sites from 15 countries. Solve-RD is built upon a core group of four European Reference Networks (ERNs; ERN-ITHACA, ERN-RND, ERN-Euro NMD, ERN-GENTURIS) which annually see more than 270,000 RD patients with respective pathologies. The main ambition is to solve unsolved rare diseases for which a molecular cause is not yet known. This is achieved through an innovative clinical research environment that introduces novel ways to organise expertise and data. Two major approaches are being pursued (i) massive data re-analysis of >19,000 unsolved rare disease patients and (ii) novel combined -omics approaches. The minimum requirement to be eligible for the analysis activities is an inconclusive exome that can be shared with controlled access. The first preliminary data re-analysis has already diagnosed 255 cases form 8393 exomes/genome datasets. This unprecedented degree of collaboration focused on sharing of data and expertise shall identify many new disease genes and enable diagnosis of many so far undiagnosed patients from all over Europe.

Rare Diseases (RD) are individually rare but collectively a common health issue. Around 80% of RD are estimated to have a genetic cause [1]. The time to a genetic diagnosis however often takes several years and initial clinical diagnoses are incorrect in up to 40% of families [2]. Around 50% of patients with a RD remain undiagnosed even in advanced expert clinical settings where whole exome sequencing (WES) is applied routinely as a diagnostic approach. Depending on the exact diagnostic setting, the inclusion criteria and the type of RD, the diagnostic yield from WES ranges between 15 and 51% of cases [3, 4].

At least two scenarios allow boosting the current yield of WES. Firstly, there is a value in re-analysing WES data regularly [5] and on massive scale [6], but not every RD expert has access to tools enabling this systematically. Secondly, it is clear that moving beyond the exome can provide additional benefits [7, 8].

Solve-RD aims to solve a large number of unsolved RD, for which a molecular cause is not yet known, by implementing both strategies mentioned above. To this end, Solve-RD applies innovative ways to effectively organise expertise and data.

## Cohorts

To structure its work Solve-RD has defined four types of *cohorts*. *Cohort 1*, “Unsolved Cases”, comprises cases with an inconclusive WES or whole genome sequencing (WGS) from any partnering or associated ERN center. These data undergo a comprehensive re-analysis effort. *Cohort 2*, “Specific ERN Cohorts”, represent disease group specific ERN cohorts that are analysed by newly applied tailored -omics approaches. *Cohort 3*, “Ultra-Rare Rare Diseases”, includes (groups of) patients with unique phenotypes identified (and matched) by RD experts from all ERN participants. For the diseases included in *Cohort 4*, “The Unsolvables”, all relevant -omics methodologies will be used to solve highly recognisable, clinically well-defined disease entities for which the disease cause has not been found yet despite considerable previous research investigations including WES and WGS (Table 1).

**Table 1:** Examples for the specific ERN cohorts and the unsolvables.

Cohort	Rationale
*Cohort 2: Long-read whole genome sequencing (LR-WGS)*	
X-linked spinal and bulbar muscular atrophy (SBMA)	Suspected expansions of repeat disorder or other hidden structural variants (SV)
Hereditary ataxia	Suspected expansions of repeat disorder or other hidden SVs
*Cohort 2: Genomics and Epigenomics*	
Unexplained Intellectual Disability (ID): patient-parent trios	De novo mutation prioritisation very powerful filter for de novo methylation changes
Diffuse gastric cancer	Hypermethylation of cancer gene promoter known disease mechanism
Rare pheochromocytomas and paragangliomas	Hypermethylation of cancer gene promoter known disease mechanism
*Cohort 4*	
Unsolved syndromes available via ERN ITHACA	Aicardi syndrome, Gomez–Lopez Hernandes syndrome, Hallermann–Streiff syndrome are clinically well-defined entities and have been studied by WES and WGS globally and remain unsolved

In total, Solve-RD is targeting to re-analyse >19,000 datasets for cohort 1, sequence ~3500 short- and long-read WGS for cohorts 2, 3, and 4 and add >3500 additional -omics experiments including RNA sequencing, epigenomics, metabolomics, Deep-WES, and deep molecular phenotyping. Data collected and produced in Solve-RD shall be shared via the European Genome-Phenome Archive (EGA) and the RD-Connect Genome-Phenome Analysis Platform (GPAP) to allow controlled access by other RD initiatives and scientists.

## Organisation of data

**Fig. 1:**
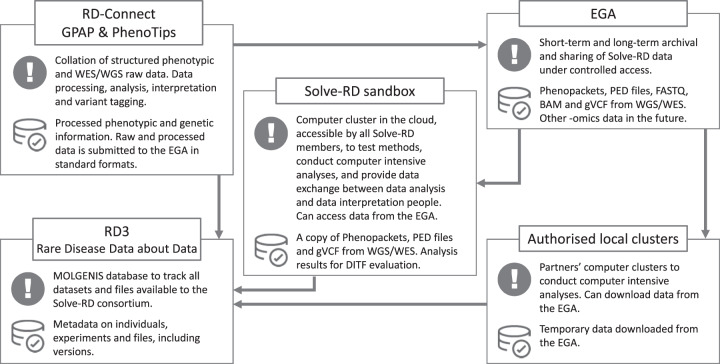
Solve-RD data infrastructure. Key components of the Solve-RD infrastructure for multi-omics data analysis, illustrating main use and data available.

The Solve-RD strategy relies on the availability of large amounts of good quality, standardised genomic and phenotypic data and metadata from undiagnosed RD patients and their relatives. Solve-RD follows a centralised approach, to enable all envisioned analyses. Data sharing in Solve-RD is regulated by policy documents, available on the project’s website. To overcome the technical challenge of centralising large amounts of data, Solve-RD leverages existing infrastructures such as EGA, GPAP, and computing clusters from project partners (Fig. 1). In addition, Solve-RD is developing a cloud-based computing cluster for collaborative analysis and methods testing (the Solve-RD Sandbox) and a central database to control and view all the project’s data and metadata (RD3; rare disease data about data) using the MOLGENIS open source data platform [9]. Clinical data and pedigree structure for all participating individuals is collated through standard terms and ontologies such as HPO, ORDO, and OMIM using GPAP-PhenoStore. To share data within the project and beyond, Solve-RD is an early adopter of the recently GA4GH-approved (Global Alliance for Genomics and Health, https://www.ga4gh.org) PhenoPackets standard to enable exchange of phenotypic and family information [10].

For each individual, WES and/or WGS data are submitted to GPAP in FASTQ, BAM, or CRAM format. The sequencing data are processed through a standard pipeline based on GATK (Genomic Analysis Toolkit variant calling software) best practices [11, 12]. After that, PhenoPackets, PED files (for pedigrees), raw data (FASTQ), alignments (BAM) and genetic variants (gVCF) are transferred to the EGA, where they are archived and made available to the consortium (and later on to the broader RD community) for further analysis. Furthermore, Solve-RD data are connected to MatchMaker Exchange via GPAP.

To reach the ambitious goal to collect 19,000 unsolved WES/WGS, Solve-RD has defined several deadlines to submit data to the project. After each deadline, all data are processed and released as a data freeze, which is amenable to corrections via patches. The first data freeze, released in early 2020, includes data from 8,393 individuals.

In parallel to the collection of existing data for cohort 1, new omics data are being generated for cohorts 2, 3, and 4. A common data workflow has been established for all these data types (Fig. 1). The data collated and generated by Solve-RD constitutes a unique collection that will be valuable beyond the project, and the consortium is committed to make it FAIR under controlled access, through the EGA and GPAP.

## Organisation of expertise

Solve-RD works on the interphase of many disciplines relevant to solving the unsolved RD. Central to the RD field are clinical geneticists and clinical scientists organised in the respective ERNs. Solve-RD provides expertise in genomics and other -omics data analysis, through data scientists, molecular geneticists, and bioinformaticians.

**Fig. 2:**
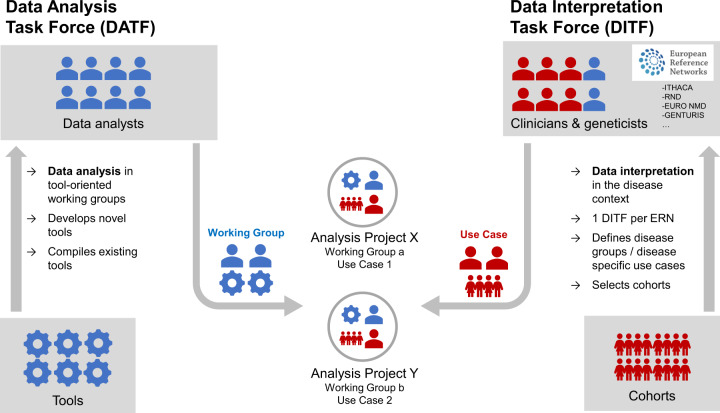
The Solve-RD data analysis structure ‘in action’. Consisting of the Data Analysis Task Force (DATF) and four Data Interpretation Task Forces (DITF)—one per core ERN involved. The DATF established working groups (WGs) for specific analyses. Working groups and DITFs jointly work on analysis projects based on use cases described by the DITF members.

To warrant the best exchange of expertise we have implemented two structures: (i) Data scientists and genomics experts are organised in a Data Analysis Task Force (DATF), (ii) Expert clinicians and geneticists from each ERN are organised in a Data Interpretation Task Force (DITF) (Fig. 2). The tasks for these structures are in brief: ►DITF: define needs of ERN for (a) data re-analysis and (b) novel -omics data; define use cases for re-analysis and novel analysis; discuss/test suitable data output formats for clinical scientists; coordinate collaborative data interpretation; discuss within respective ERN network and feedback to DATF. ►DATF: map expertise in Solve-RD and all (ERN-)partners; create *Analysis Projects* (Supplementary Table S1) based on ERNs needs; develop state-of-the-art analysis tools; analyse data: (a) data re-analysis and (b) novel omics data; optimise data sharing and output formats for DITF/ERNs.

**Fig. 3:**
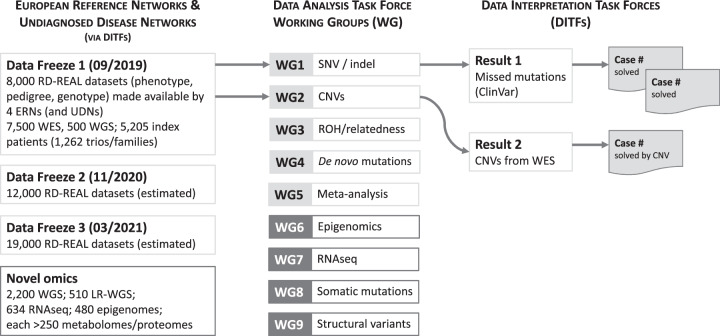
Organisation of new result flow in Solve-RD. Working groups (WG) 1–5 will re-analyse existing sequencing data. Novel omics data will be analysed by all working groups (as appropriate). RD-REAL refers to Rare Disease - REAnalysis Logistics.

The structure implemented for data re-analysis has proven efficient and versatile [13], and will therefore be applied for novel omics data analysis, with additional working groups for specific -omics technologies (Fig. 3).

To integrate expertise not available within the Solve-RD consortium, particularly with regards to molecular and functional validation of newly found genes, Solve-RD is implementing an innovative brokerage system (Rare Disease Models and Mechanisms Network—Europe (RDMM-Europe)) that has already been successfully used in Canada [14]. As of 4 December 2020, 14 “brokering” Seeding Grants have been awarded to external model investigators.

## Achievements and challenges

The work of the first 3 years of Solve-RD resulted in a practical solution to share and jointly analyse 8393 datasets from all over Europe: Solve-RD organised RD expertise via a DITF and DATF with the respective working group structure described above. The first re-analysis approaches resulted in 255 newly diagnosed cases, mainly by leveraging latest ClinVar entries. As examples we refer to adjacent articles, published jointly in this issue [13, 15–18]. Many more candidate variants and new analysis results are under evaluation.

To achieve its current status Solve-RD has successfully addressed some critical challenges that are (a) European data sharing in accordance with GDPR, (b) heterogeneity in existing WES data (e.g. 26 WES kits so far; multiple sequencing platforms), (c) implementing a centralised analysis approach and (d) addressing the rarity of events.

It is the vision of Solve-RD that, by the end of the project, the Solve-RD dataset will be the largest well-annotated, standardised, multi-omics RD dataset on the diseases covered by the four core ERNs. In this sense, we hope that the Solve-RD dataset will be as useful to the RD community as the gnomAD consortium is for the genomics community [19], by making -omics data of RD populations available to the community.

## Supplementary information

Supplementary Information

Supplementary Table S1

Solve-RD consortium author list
