# Supplementary material for: Solve-RD: systematic pan-European data sharing and collaborative analysis to solve rare diseases
Source: Eur J Hum Genet. 2021 Jun 1;29(9):1325–31. doi: 10.1038/s41431-021-00859-0 (PMC8440542; doi:10.1038/s41431-021-00859-0)
Supplement: Supplementary file 1 — Supplementary Information [file 41431_2021_859_MOESM1_ESM.docx]

**Supplementary Information**

Zurek B, Ellwanger K, Vissers L, Schüle R, Synofzik M, Töpf A, et al. Solve-RD: systematic Pan- European data sharing and collaborative analysis to solve Rare Diseases.

Details on Solve-RD Working groups

The “SNV/indel” WG (WG1) is using the GPAP APIs (Application Programming Interfaces) with different combinations of filters to programmatically re-analyse the exome/genome-phenome data. The initial approach to discover the most obvious cases (“low-hanging fruit”) has contributed to solve 120 cases (1). This WG has also combined and annotated all the gVCFs with a common pipeline to make them available through the Sandbox for further analyses. gVCF files, together with PhenoPackets, are used as input for Exomiser (2, 3), LIRICAL (4) and CAPICE (5).

The “Copy Number Variation (CNV)” WG (WG2) has already processed all existing WES data. Subclusters for experiments batched by the enrichment kit used for library preparation were generated by ClusterWES (6). Each of these 85 clusters was then used for CNV detection by four tools: VarGenius (7), ClinCNV (<https://github.com/imgag/ClinCNV>), Conifer (8) and ExomeDepth (9).

The “Runs of Homozygosity (ROH) and relatedness” WG3 has performed quality control tests on the dataset using several methods in parallel, including kinship analyses and the estimation of consanguinity based on the computation of ROH as recently described (10).

The “*de novo* mutations” WG4 have analysed trios to systematically identify *de novo* mutations using existing and novel tools. Finally, the “Meta-analysis” WG5 aims to identify genes with an excess of variants i.e. ‘variant burden’ in certain patient cohorts, with the first candidate genes already being evaluated.

**Supplementary References**

1. Matalonga L, Hernández-Ferrer C, Piscia D, Solve-RD SNV-indel working group, Vissers LELM, Schüle R, et al. Diagnosis of rare disease patients through programmatic reanalysis of genome-phenome data. Manuscript submitted to EJHG.

2. Smedley D, Jacobsen JO, Jäger M, Köhler S, Holtgrewe M, Schubach M, et al. Next-generation diagnostics and disease-gene discovery with the Exomiser. Nat Protoc. 2015;10(12):2004-15.

3. Robinson PN, Köhler S, Oellrich A, Wang K, Mungall CJ, Lewis SE, et al. Improved exome prioritization of disease genes through cross-species phenotype comparison. Genome Res. 2014;24(2):340-8.

4. Robinson PN, Ravanmehr V, Jacobsen JOB, Danis D, Zhang XA, Carmody LC, et al. Interpretable Clinical Genomics with a Likelihood Ratio Paradigm. Am J Hum Genet. 2020;107(3):403-17.

5. Li S, van der Velde KJ, de Ridder D, van Dijk ADJ, Soudis D, Zwerwer LR, et al. CAPICE: a computational method for Consequence-Agnostic Pathogenicity Interpretation of Clinical Exome variations. Genome Med. 2020;12(1):75.

6. Johansson L. Manuscript in preparation2020.

7. Musacchia F, Ciolfi A, Mutarelli M, Bruselles A, Castello R, Pinelli M, et al. VarGenius executes cohort-level DNA-seq variant calling and annotation and allows to manage the resulting data through a PostgreSQL database. BMC Bioinformatics. 2018;19(1):477.

8. Krumm N, Sudmant PH, Ko A, O'Roak BJ, Malig M, Coe BP, et al. Copy number variation detection and genotyping from exome sequence data. Genome Res. 2012;22(8):1525-32.

9. Plagnol V, Curtis J, Epstein M, Mok KY, Stebbings E, Grigoriadou S, et al. A robust model for read count data in exome sequencing experiments and implications for copy number variant calling. Bioinformatics. 2012;28(21):2747-54.

10. Matalonga L, Laurie S, Papakonstantinou A, Piscia D, Mereu E, Bullich G, et al. Improved Diagnosis of Rare Disease Patients through Systematic Detection of Runs of Homozygosity. J Mol Diagn. 2020.
