# Supplementary material for: Solve-RD: systematic pan-European data sharing and collaborative analysis to solve rare diseases
Source: Eur J Hum Genet. 2021 Jun 1;29(9):1325–31. doi: 10.1038/s41431-021-00859-0 (PMC8440542; doi:10.1038/s41431-021-00859-0)
Supplement: Supplementary file 2 — Supplementary Table S1 [file 41431_2021_859_MOESM2_ESM.docx]

**Solve-RD analysis projects for existing exome and genome data**

| Title | DITF | WG |
| --- | --- | --- |
| Solve-RD QC analysis: Perform quality control on all Solve-RD samples | All | Several WGs |
| Solve-RD ancestry and admixture analysis: For all Solve-RD samples, determine ancestry and admixture | All | Several WGs |
| Solve-RD variant annotation: Annotate all Solve-RD samples using multiple databases | All | WG1 SNV/indel |
| Automated SNV and Indel filtering and prioritisation | All | WG1 SNV/indel |
| Prevalence of pathogenic variant in genes associated in with known tumour risk syndromes | GENTURIS | WG1 SNV/indel & WG5 Meta-analysis |
| Detection of mitochondrial DNA variants from WES/WGS | All | WG1 SNV/indel |
| Landscape of rare genetic variants in titin gene | EURO NMD | WG1 SNV/indel |
| Clinvar class IV-V mutational burden analysis of exome negative (unsolved) patients with intellectual disability (ID) | ITHACA | WG1 SNV/indel & WG5 Meta-analysis |
| Detection of Copy Number Variants in WES and WGS data experiment in the Solve-RD re-analysis cohort | All | WG2 CNV |
| Solve-RD CNV analysis using Conifer: detect possible copy-number variations using multiple CNV tools | All | WG2 CNV |
| Identification and Interpretation of rare structural variants in WES-based rare-disease diagnostics | All | WG2 CNV |
| Solve-RD STR analysis: detect aberrant short tandem repeats | All | WG2 CNV |
| Solve-RD UPD analysis: detect possible uniparental disomies | All | WG2 CNV |
| Run of homozygosity, consanguinity, relatedness and ancestry analysis | All | WG3 RoH / relatedness |
| Solve-RD de novo variant calling in patient-parent trios | All | WG4 *De novo* mutations |
| Solve-RD meta-analysis: Compare case and control cohorts to find novel disease genes based on statistical enrichment and overlap analysis | All | WG5 Meta-analysis |
