# Supplementary material for: Solve-RD: systematic pan-European data sharing and collaborative analysis to solve rare diseases
Source: Eur J Hum Genet. 2021 Jun 1;29(9):1325–31. doi: 10.1038/s41431-021-00859-0 (PMC8440542; doi:10.1038/s41431-021-00859-0)
Supplement: Supplementary file 3 — Solve-RD consortium author list [file 41431_2021_859_MOESM3_ESM.docx]

**Solve-RD consortium* author list**

Olaf Riess^[[1]](#endnote-1),^^[[2]](#endnote-2)^, Tobias B. Haack1 , Holm Graessner1^,^2, Birte Zurek1^,^2, Kornelia Ellwanger1^,^2, Stephan Ossowski1, German Demidov1, Marc Sturm1, Julia M. Schulze-Hentrich1, Rebecca Schüle^[[3]](#endnote-3)^^,^^[[4]](#endnote-4)^, Christoph Kessler3^,^4 , Melanie Wayand3^,^4, Matthis Synofzik3^,^4, Carlo Wilke3^,^4, Andreas Traschütz3^,^4, Ludger Schöls3^,^4, Holger Hengel3^,^4, Peter Heutink3^,^4, Han Brunner^[[5]](#endnote-5),^^[[6]](#endnote-6),^^[[7]](#endnote-7)^, Hans Scheffer5^,^6, Nicoline Hoogerbrugge5^,^^[[8]](#endnote-8)^ , Alexander Hoischen5^,^8^,^^[[9]](#endnote-9)^, Peter A.C. ’t Hoen^8,^^[[10]](#endnote-10)^ ,Lisenka E.L.M. Vissers5^,^7, Christian Gilissen5^,^8, Wouter Steyaert5^,^8, Karolis Sablauskas5, Richarda M. de Voer5^,^8, Erik-Jan Kamsteeg5, Bart van de Warrenburg7^,^^[[11]](#endnote-11)^, Nienke van Os7^,11^ , Iris te Paske5^,^8, Erik Janssen5^,^8, Elke de Boer5^,^7, Marloes Steehouwer5, Burcu Yaldiz5, Tjitske Kleefstra5^,^7, Anthony J. Brookes^[[12]](#endnote-12)^, Colin Veal^12^, Spencer Gibson^12^, Marc Wadsley^12^, Mehdi Mehtarizadeh^12^, Umar Riaz^12^, Greg Warren^12^, Farid Yavari Dizjikan^12^, Thomas Shorter^12,^ Ana Töpf^[[13]](#endnote-13)^, Volker Straub^13^, Chiara Marini Bettolo^13^, Sabine Specht^13^, Jill Clayton-Smith^[[14]](#endnote-14)^, Siddharth Banka14^,^^[[15]](#endnote-15)^, Elizabeth Alexander14, Adam Jackson14, Laurence Faivre^[[16]](#endnote-16),^^[[17]](#endnote-17),^^[[18]](#endnote-18),^^[[19]](#endnote-19),^^[[20]](#endnote-20)^ Christel Thauvin17^,^18^,^19^,^20^,20^, Antonio Vitobello18, Anne-Sophie Denommé-Pichon18, Yannis Duffourd^18,19^, Emilie Tisserant^18^, Ange-Line Bruel^18^, Christine Peyron^[[21]](#endnote-21),^^[[22]](#endnote-22)^, Aurore Pélissier22^,22^, Sergi Beltran^[[23]](#endnote-23),^^[[24]](#endnote-24)^, Ivo Glynne Gut24^,24^, Steven Laurie24, Davide Piscia24, Leslie Matalonga24, Anastasios Papakonstantinou24, Gemma Bullich24, Alberto Corvo24, Carles Garcia24, Marcos Fernandez-Callejo24, Carles Hernández24, Daniel Picó24, Ida Paramonov24, Hanns Lochmüller24, Gulcin Gumus^[[25]](#endnote-25)^, Virginie Bros-Facer^[[26]](#endnote-26)^, Ana Rath^[[27]](#endnote-27)^, Marc Hanauer27, Annie Olry27, David Lagorce27, Svitlana Havrylenko27, Katia Izem27, Fanny Rigour27, Giovanni Stevanin^[[28]](#endnote-28),^^[[29]](#endnote-29),^^[[30]](#endnote-30),^^[[31]](#endnote-31),^^[[32]](#endnote-32)^, Alexandra Durr29^,^30^,^31^, 31,^^[[33]](#endnote-33)^, Claire-Sophie Davoine29^,^30^,^31^,^32^,32^, Léna Guillot-Noel29^,^30^,^31^,^32^,32^, Anna Heinzmann29^,^30^,^31^,31,^^[[34]](#endnote-34)^, Giulia Coarelli29^,^30^,^31^,31,34^, Gisèle Bonne^[[35]](#endnote-35)^, Teresinha Evangelista^35^, Valérie Allamand^35^, Isabelle Nelson^35^, Rabah Ben Yaou^35,^^[[36]](#endnote-36),^^[[37]](#endnote-37)^, Corinne Metay^35,^^[[38]](#endnote-38)^, Bruno Eymard^35,^36, Enzo Cohen^35^, Antonio Atalaia^35^, Tanya Stojkovic^35,^36, Milan Macek Jr.^[[39]](#endnote-39)^, Marek Turnovec^39^, Dana Thomasová^39^, Radka Pourová Kremliková^39^, Vera Franková^39^, Markéta Havlovicová^39^, Vlastimil Kremlik^39^, Helen Parkinson^[[40]](#endnote-40)^, Thomas Keane^40^, Dylan Spalding^40^, Alexander Senf^40^, Peter Robinson^[[41]](#endnote-41)^, Daniel Danis^41^, Glenn Robert^[[42]](#endnote-42)^, Alessia Costa^42^42, Christine Patch^42^42^,^^[[43]](#endnote-43)^, Mike Hanna^[[44]](#endnote-44)^, Henry Houlden^[[45]](#endnote-45)^, Mary Reilly44, Jana Vandrovcova45, Francesco Muntoni^[[46]](#endnote-46),^^[[47]](#endnote-47)^, Irina Zaharieva46, Anna Sarkozy46, Vincent Timmerman^[[48]](#endnote-48),^^[[49]](#endnote-49)^, Jonathan Baets^[[50]](#endnote-50),^^[[51]](#endnote-51),^^[[52]](#endnote-52)^, Liedewei Van de Vondel49^,^50, Danique Beijer49^,^50, Peter de Jonghe49^,^51, Vincenzo Nigro^[[53]](#endnote-53),^^[[54]](#endnote-54)^, Sandro Banfi53^,^54, Annalaura Torella53, Francesco Musacchia53^,^54, Giulio Piluso53, Alessandra Ferlini^[[55]](#endnote-55)^, Rita Selvatici55, Rachele Rossi55, Marcella Neri55, Stefan Aretz^[[56]](#endnote-56),^^[[57]](#endnote-57)^, Isabel Spier56^,^57, Anna Katharina Sommer56, Sophia Peters56, Carla Oliveira^[[58]](#endnote-58),^^[[59]](#endnote-59),^^[[60]](#endnote-60)^, Jose Garcia Pelaez58^,^59, Ana Rita Matos58^,^59, Celina São José58^,^59, Marta Ferreira58^,^59, Irene Gullo58^,^59^,^60, Susana Fernandes58^,^^[[61]](#endnote-61)^, Luzia Garrido^[[62]](#endnote-62)^, Pedro Ferreira58^,^59^,^^[[63]](#endnote-63)^, Fátima Carneiro58^,^59^,^60, Morris A. Swertz^[[64]](#endnote-64)^, Lennart Johansson^64^, Joeri K. van der Velde^64^, Gerben van der Vries^64^, Pieter B. Neerincx^64^, Dieuwke Roelofs-Prins^64^, Sebastian Köhler^[[65]](#endnote-65)^, Alison Metcalfe^42,^^[[66]](#endnote-66)^, Alain Verloes^[[67]](#endnote-67),^^[[68]](#endnote-68)^, Séverine Drunat^67,68^, Caroline Rooryck^[[69]](#endnote-69)^, Aurelien Trimouille^[[70]](#endnote-70)^, Raffaele Castello54, Manuela Morleo54, Michele Pinelli54, Alessandra Varavallo54, Manuel Posada De la Paz^[[71]](#endnote-71)^, Eva Bermejo Sánchez^71^, Estrella López Martín^71^, Beatriz Martínez Delgado^71^, F. Javier Alonso García de la Rosa^71^, Andrea Ciolfi^[[72]](#endnote-72)^, Bruno Dallapiccola^72^, Simone Pizzi^72^, Francesca Clementina Radio^72^, Marco Tartaglia^72^, Alessandra Renieri^[[73]](#endnote-73),^^[[74]](#endnote-74),^^[[75]](#endnote-75)^, Elisa Benetti^73^, Peter Balicza^[[76]](#endnote-76)^, Maria Judit Molnar^76^, Ales Maver^[[77]](#endnote-77)^, Borut Peterlin^77^, Alexander Münchau^[[78]](#endnote-78)^, Katja Lohmann^78^, Rebecca Herzog^78^, Martje Pauly^78^, Alfons Macaya^[[79]](#endnote-79)^, Anna Marcé-Grau^79^, Andres Nascimiento Osorio^[[80]](#endnote-80)^, Daniel Natera de Benito^80^, Hanns Lochmüller^[[81]](#endnote-81),^^[[82]](#endnote-82),^^[[83]](#endnote-83)^, Rachel Thompson^81,83^, Kiran Polavarapu^81^, David Beeson^[[84]](#endnote-84)^, Judith Cossins^84^, Pedro M. Rodriguez Cruz^84^, Peter Hackman^[[85]](#endnote-85)^, Mridul Johari^85^, Marco Savarese^85^, Bjarne Udd^85,^^[[86]](#endnote-86),^^[[87]](#endnote-87)^, Rita Horvath^[[88]](#endnote-88)^, Gabriel Capella^[[89]](#endnote-89)^, Laura Valle^89^, Elke Holinski-Feder^[[90]](#endnote-90)^, Andreas Laner^90^, Verena Steinke-Lange^90^, Evelin Schröck^[[91]](#endnote-91)^, Andreas Rump^91^,^[[92]](#endnote-92)^,

1. Institute of Medical Genetics and Applied Genomics, University of Tübingen, Tübingen, Germany. [↑](#endnote-ref-1)
2. Centre for Rare Diseases, University of Tübingen, Tübingen, Germany. [↑](#endnote-ref-2)
3. Department of Neurodegeneration, Hertie Institute for Clinical Brain Research (HIH), University of Tübingen, Tübingen, Germany. [↑](#endnote-ref-3)
4. German Center for Neurodegenerative Diseases (DZNE), Tübingen, Germany. [↑](#endnote-ref-4)
5. Department of Human Genetics, Radboud University Medical Center, Nijmegen, The Netherlands. [↑](#endnote-ref-5)
6. Department of Clinical Genetics, Maastricht University Medical Centre, Maastricht, The Netherlands. [↑](#endnote-ref-6)
7. Donders Institute for Brain, Cognition and Behaviour, Radboud University Medical Center, Nijmegen, The Netherlands. [↑](#endnote-ref-7)
8. Radboud Institute for Molecular Life Sciences, Nijmegen, the Netherlands. [↑](#endnote-ref-8)
9. Department of Internal Medicine and Radboud Center for Infectious Diseases (RCI), Radboud University Medical Center, Nijmegen, The Netherlands. [↑](#endnote-ref-9)
10. Center for Molecular and Biomolecular Informatics, Radboud university medical center, Nijmegen, The Netherlands. [↑](#endnote-ref-10)
11. Department of Neurology, Radboud University Medical Center, Nijmegen, The Netherlands. [↑](#endnote-ref-11)
12. Department of Genetics and Genome Biology, University of Leicester, Leicester, UK. [↑](#endnote-ref-12)
13. John Walton Muscular Dystrophy Research Centre, Translational and Clinical Research Institute, Newcastle University and Newcastle Hospitals NHS Foundation Trust, Newcastle upon Tyne, UK. [↑](#endnote-ref-13)
14. Division of Evolution and Genomic Sciences, School of Biological Sciences, Faculty of Biology, Medicine and Health, University of Manchester, Manchester M13 9WL, UK. [↑](#endnote-ref-14)
15. Manchester Centre for Genomic Medicine, St Mary's Hospital, Manchester University Hospitals NHS Foundation Trust, Health Innovation Manchester, Manchester M13 9WL, UK. [↑](#endnote-ref-15)
16. Dijon University Hospital, Genetics Department, Dijon, France. [↑](#endnote-ref-16)
17. Dijon University Hospital, Centre of Reference for Rare Diseases: Development disorders and malformation syndromes, Dijon, France. [↑](#endnote-ref-17)
18. Inserm - University of Burgundy-Franche Comté, UMR1231 GAD, Dijon, France. [↑](#endnote-ref-18)
19. Dijon University Hospital, FHU-TRANSLAD, Dijon, France. [↑](#endnote-ref-19)
20. Dijon University Hospital, GIMI institute, Dijon, France. [↑](#endnote-ref-20)
21. University of Burgundy-Franche Comté, Dijon Economics Laboratory, Dijon, France. [↑](#endnote-ref-21)
22. University of Burgundy-Franche Comté, FHU-TRANSLAD, Dijon, France. [↑](#endnote-ref-22)
23. CNAG‐CRG, Centre for Genomic Regulation (CRG), The Barcelona Institute of Science and Technology, Baldiri Reixac 4, Barcelona 08028, Spain. [↑](#endnote-ref-23)
24. Universitat Pompeu Fabra (UPF), Barcelona, Spain. [↑](#endnote-ref-24)
25. EURORDIS-Rare Diseases Europe, Sant Antoni Maria Claret 167 - 08025 Barcelona, Spain. [↑](#endnote-ref-25)
26. EURORDIS-Rare Diseases Europe, Plateforme Maladies Rares, 75014 Paris, France. [↑](#endnote-ref-26)
27. INSERM, US14 - Orphanet, Plateforme Maladies Rares, 75014 Paris, France. [↑](#endnote-ref-27)
28. Institut National de la Santé et de la Recherche Medicale (INSERM) U1127, Paris, France. [↑](#endnote-ref-28)
29. Centre National de la Recherche Scientifique, Unité Mixte de Recherche (UMR) 7225, Paris, France. [↑](#endnote-ref-29)
30. Unité Mixte de Recherche en Santé 1127, Université Pierre et Marie Curie (Paris 06), Sorbonne Universités, Paris, France. [↑](#endnote-ref-30)
31. Institut du Cerveau -ICM, Paris, France. [↑](#endnote-ref-31)
32. Ecole Pratique des Hautes Etudes, Paris Sciences et Lettres Research University, Paris, France. [↑](#endnote-ref-32)
33. Centre de Référence de Neurogénétique, Hôpital de la Pitié-Salpêtrière, Assistance Publique-Hôpitaux de Paris (AP-HP), Paris, France. [↑](#endnote-ref-33)
34. Hôpital de la Pitié-Salpêtrière, Assistance Publique-Hôpitaux de Paris (AP-HP), Paris, France. [↑](#endnote-ref-34)
35. Sorbonne Université, INSERM UMRS_974, Center of Research in Myology, 75013 Paris, France. [↑](#endnote-ref-35)
36. AP-HP, Centre de Référence de Pathologie Neuromusculaire Nord, Est, Ile-de-France, Institut de Myologie, G.H. Pitié-Salpêtrière, F-75013 Paris, France. [↑](#endnote-ref-36)
37. Institut de Myologie, Equipe Bases de données, G.H. Pitié-Salpêtrière, F-75013 Paris, France. [↑](#endnote-ref-37)
38. AP-HP, Unité Fonctionnelle de Cardiogénétique et Myogénétique Moléculaire et Cellulaire, G.H. Pitié-Salpêtrière, F-75013 Paris, France. [↑](#endnote-ref-38)
39. Department of Biology and Medical Genetics, Charles University Prague-2nd Faculty of Medicine and University Hospital Motol, Prague, Czech Republic. [↑](#endnote-ref-39)
40. European Bioinformatics Institute, European Molecular Biology Laboratory, Wellcome Genome Campus, Hinxton, Cambridge, United Kingdom. [↑](#endnote-ref-40)
41. Jackson Laboratory for Genomic Medicine, Farmington, CT 06032, USA. [↑](#endnote-ref-41)
42. Florence Nightingale Faculty of Nursing and Midwifery, King's College, London, UK. [↑](#endnote-ref-42)
43. Genetic Counselling, Genomics England, Queen Mary University of London, Dawson Hall, EC1M 6BQ, London. [↑](#endnote-ref-43)
44. MRC Centre for Neuromuscular Diseases and National Hospital for Neurology and Neurosurgery, UCL Queen Square Institute of Neurology, London, UK. [↑](#endnote-ref-44)
45. Department of Neuromuscular Diseases, UCL Queen Square Institute of Neurology, London, UK. [↑](#endnote-ref-45)
46. Dubowitz Neuromuscular Centre, UCL Great Ormond Street Hospital, London, UK. [↑](#endnote-ref-46)
47. NIHR Great Ormond Street Hospital Biomedical Research Centre, London, United Kingdom. [↑](#endnote-ref-47)
48. Peripheral Neuropathy Research Group, Department of Biomedical Sciences, University of Antwerp, Antwerp, Belgium. [↑](#endnote-ref-48)
49. Institute Born Bunge, Antwerp, Belgium. [↑](#endnote-ref-49)
50. Peripheral Neuropathy Research Group, University of Antwerp, Antwerp, Belgium. [↑](#endnote-ref-50)
51. Neuromuscular Reference Centre, Department of Neurology, Antwerp University Hospital, Antwerpen, Belgium. [↑](#endnote-ref-51)
52. Laboratory of Neuromuscular Pathology, Institute Born-Bunge, University of Antwerp, Antwerpen, Belgium. [↑](#endnote-ref-52)
53. Dipartimento di Medicina di Precisione, Università degli Studi della Campania "Luigi Vanvitelli," Napoli, Italy. [↑](#endnote-ref-53)
54. Telethon Institute of Genetics and Medicine, Pozzuoli, Italy. [↑](#endnote-ref-54)
55. Unit of Medical Genetics, Department of Medical Sciences, University of Ferrara, Italy. [↑](#endnote-ref-55)
56. Institute of Human Genetics, University of Bonn, Bonn, Germany. [↑](#endnote-ref-56)
57. Center for Hereditary Tumor Syndromes, University Hospital Bonn, Bonn, Germany. [↑](#endnote-ref-57)
58. i3S - Instituto de Investigação e Inovação em Saúde, Universidade do Porto, Portugal. [↑](#endnote-ref-58)
59. IPATIMUP - Institute of Molecular Pathology and Immunology of the University of Porto, Portugal. [↑](#endnote-ref-59)
60. Departament of Pathology, Faculty of Medicine, University of Porto, Portugal. [↑](#endnote-ref-60)
61. Departament of Genetics, Faculty of Medicine, University of Porto, Portugal. [↑](#endnote-ref-61)
62. CHUSJ, Centro Hospitalar e Universitário de São João, Porto, Portugal. [↑](#endnote-ref-62)
63. Faculty of Sciences, University of Porto, Portugal. [↑](#endnote-ref-63)
64. Department of Genetics, Genomics Coordination Center, University Medical Center Groningen, University of Groningen, Groningen, The Netherlands. [↑](#endnote-ref-64)
65. NeuroCure Cluster of Excellence, Charité Universitätsklinikum, Charitéplatz 1, 10117 Berlin, Germany. [↑](#endnote-ref-65)
66. College of Health, Well-being and Life-Sciences, Sheffield Hallam University, Sheffield, UK. [↑](#endnote-ref-66)
67. Dept of Genetics, Assistance Publique-Hôpitaux de Paris - Université de Paris, Robert DEBRE University Hospital, 48 bd SERURIER, Paris, France. [↑](#endnote-ref-67)
68. INSERM UMR 1141 "NeuroDiderot", Hôpital R DEBRE, Paris, France. [↑](#endnote-ref-68)
69. Univ. Bordeaux, MRGM INSERM U1211, CHU de Bordeaux, Service de Génétique Médicale , F-33000 Bordeaux, France. [↑](#endnote-ref-69)
70. Laboratoire de Génétique Moléculaire, Service de Génétique Médicale, CHU Bordeaux – Hôpital Pellegrin, Place Amélie Raba Léon, 33076 Bordeaux Cedex, France. [↑](#endnote-ref-70)
71. Institute of Rare Diseases Research, Spanish Undiagnosed Rare Diseases Cases Program (SpainUDP) & Undiagnosed Diseases Network International (UDNI), Instituto de Salud Carlos III, Madrid, Spain. [↑](#endnote-ref-71)
72. Genetics and Rare Diseases Research Division, Ospedale Pediatrico Bambino Gesù, IRCCS, 00146 Rome, Italy. [↑](#endnote-ref-72)
73. Med Biotech Hub and Competence Center, Department of Medical Biotechnologies, University of Siena, Italy. [↑](#endnote-ref-73)
74. Medical Genetics, University of Siena, Italy. [↑](#endnote-ref-74)
75. Genetica Medica, Azienda Ospedaliero-Universitaria Senese, Italy. [↑](#endnote-ref-75)
76. Institute of Genomic Medicine and Rare Diseases, Semmelweis University, Budapest, Hungary. [↑](#endnote-ref-76)
77. Clinical institute of genomic medicine, University medical centre Ljubljana, Slovenia. [↑](#endnote-ref-77)
78. Institute of Neurogenetics, University of Lübeck, Lübeck, Germany. [↑](#endnote-ref-78)
79. Neurology Research Group, Vall d’Hebron Research Institute, Universitat Autònoma de Barcelona, Barcelona, Spain. [↑](#endnote-ref-79)
80. Neuromuscular Disorders Unit , Department of Pediatric Neurology. Hospital Sant Joan de Déu, Barcelona, Spain. [↑](#endnote-ref-80)
81. Department of Neuropediatrics and Muscle Disorders, Medical Center, Faculty of Medicine, University of Freiburg, Freiburg, Germany. [↑](#endnote-ref-81)
82. Centro Nacional de Análisis Genómico (CNAG-CRG), Center for Genomic Regulation, Barcelona Institute of Science and Technology (BIST), Barcelona, Spain. [↑](#endnote-ref-82)
83. Children's Hospital of Eastern Ontario Research Institute, University of Ottawa, Ottawa, ON, Canada. [↑](#endnote-ref-83)
84. Nuffield Department of Clinical Neurosciences, University of Oxford, UK. [↑](#endnote-ref-84)
85. Folkhälsan Research Centre and Medicum, University of Helsinki, Helsinki, Finland. [↑](#endnote-ref-85)
86. Tampere Neuromuscular Center, Tampere, Finland. [↑](#endnote-ref-86)
87. Vasa Central Hospital, Vaasa, Finland. [↑](#endnote-ref-87)
88. Department of Clinical Neurosciences, University of Cambridge, Cambridge, UK. [↑](#endnote-ref-88)
89. Bellvitge Biomedical Research Institute (IDIBELL), Barcelona, Spain. [↑](#endnote-ref-89)
90. Medical Genetics Center (MGZ), Munich, Germany. [↑](#endnote-ref-90)
91. Institute for Clinical Genetics, Faculty of Medicine Carl Gustav Carus, Technical University Dresden, Dresden, Germany. [↑](#endnote-ref-91)
92. Center for Personalized Oncology, University Hospital Carl Gustav Carus, Technical University Dresden, Dresden, Germany. [↑](#endnote-ref-92)
